# Tobacco messages encountered in real-time among low socio-economic position groups: a descriptive study

**DOI:** 10.1186/s12889-021-12197-3

**Published:** 2021-11-20

**Authors:** Elise M. Stevens, Coralia Vázquez-Otero, Xiaoyan Li, Monisha Arya, Donna Vallone, Sara Minsky, Nathaniel D. Osgood, Kasisomayajula Viswanath

**Affiliations:** 1 Department of Population and Quantitative Health Sciences, Division of Preventive and Behavioral Medicine, University of Massachusetts Medical School, 368 Plantation Street, Worcester, MA 01605 USA; 2 Department of Public Health, College for Health, Community and Policy, The University of Texas at San Antonio, San Antonio, TX USA; 3 Department of Computer Science and Computational Epidemiology & Public Health Informatics Laboratory, University of Saskatchewan, Saskatoon, SK Canada; 4 Baylor College of Medicine, Houston, TX USA; 5 Truth Initiative Schroeder Institute, Washington, USA; 6 College of Global Public Health, New York University, New York City, NY USA; 7 Harvard T.H. Chan School of Public Health & Dana-Farber Cancer Institute, Harvard University, Boston, MA USA

**Keywords:** Tobacco, Advertising, Health communication

## Abstract

**Background:**

Tobacco advertising disproportionately targets low socio-economic position (SEP) groups, causing higher rates of tobacco use in this population. Anti-tobacco public health education campaigns persuade against use. This study measured real-time exposure of pro- and anti-tobacco messages from low SEP groups in two American cities.

**Methods:**

Individuals in low SEP groups (*N* = 95), aged 18–34 years old, who were smokers and non-smokers, from the Boston and Houston areas, took part in a mobile health study. They submitted images of tobacco-related messages they encountered via a mobile application for a 7-week period. Two coders analyzed the images for message characteristics. Intercoder reliability was established using Krippendorff’s alpha and data were analyzed descriptively.

**Results:**

Of the submitted images (*N* = 131), 83 were pro-tobacco and 53 were anti-tobacco. Of the pro-tobacco messages, the majority were cigarette ads (80.7%) seen outside (36.1%) or inside (30.1%) a convenience store or gas station and used conventional themes (e.g., price promotion; 53.2%). Of the anti-tobacco messages, 56.6% were sponsored by public health campaigns or were signage prohibiting smoking in a public area (39.6%). Most focused on the health harms of smoking (28.3%).

**Conclusion:**

Low SEP groups in this study encountered more pro-tobacco than anti-tobacco messages at places that were point-of-sale using price promotions to appeal to this group. Anti-tobacco messages at point-of-sale and/or advertising regulations may help combat tobacco use.

## Background

Individuals in low socio-economic position (SEP) groups use tobacco products at a higher rate [1] and suffer significantly more from tobacco-related diseases [2] than their higher socio-economic position peers. Tobacco marketing targeted specifically at low SEP groups can be one significant factor contributing to these disparities [3], while anti-tobacco messaging may be helpful in combatting tobacco use [4]. The U.S. Food and Drug Administration (FDA) has made health disparities a research priority area [5]. However, little is known about how many pro-tobacco and anti-tobacco messages low SEP groups encounter in the real world. This knowledge gap is partly because most research relies on self-reported recall of message exposure [6]. This self-reported recall can be contaminated with biases that span from respondent mood at the time of exposure [7] to how long ago they were exposed to the message [7, 8] to distance between exposure to the message and time when recall is measured. Therefore, collecting message exposure data in real-time is worthy of study [9].

Tobacco companies spend more than $9 billion per year on tobacco advertising [10, 11]. For decades, tobacco companies have disproportionately targeted low SEP groups [12], those with lower education [13], racial and ethnic minorities [14], and “blue-collar” workers [14, 15]. Communication inequality theory [16] explains this phenomenon in that there are disparities “in the generation, manipulation, and distribution of information at the group level and differences in access to and ability to take advantage of information at the individual level.” [16, 17] In other words, low SEP groups may be over-exposed to pro-tobacco messages and underexposed to anti-tobacco messages, creating disparities.

Past research has examined what types of messages low SEP groups may encounter, but most has been centered on self-report data [3], which can be contaminated with recall bias, or have been descriptive of the retail environment [12, 18–20]. These studies show that tobacco advertising is frequently found at point-of-sale locations such as gas stations and convenience stores [18–21], especially in low SEP neighborhoods [3, 20]. In addition, low SEP groups are more likely to recall tobacco advertising and report that it plays a role in whether they use the products [3], making it even more imperative to collect data about their encounters in real-time to avoid issues with time between exposure and recall. While past studies are informative, they still lack the component of what types of tobacco messaging low SEP groups are encountering in real-time.

Anti-tobacco public health campaigns have been associated with avoidance of or quitting of tobacco when undertaken in conjunction with other tobacco control policies [22, 23]. However, research shows that the actual effectiveness of public health campaigns promoting cessation among low SEP groups are rarely more effective [24]. In fact, researchers have frequently found mixed results about campaigns targeted at low SEP groups due to a myriad of factors, including possible differences in exposure to the campaigns and few campaigns targeting this population [24]. In order to be effective, campaigns must be funded and sustained and adequately funded [25].

The current study examines what low SEP individuals encounter in real-time in terms of pro- and anti-tobacco messaging, as well as the characteristics and themes of those messages to critically understand the current tobacco message environment to which they are exposed. Typical ad exposure measures can be weak due to the measures’ deep investment in time spent exposed to the message and lack of investment in specific message theme measures [26]. In the current study, using a mobile health application on a smartphone, participants reported and submitted photographs of tobacco messages that they encountered in real-time. The method of uploading the actual tobacco message has been shown to improve ad exposure measures, but has only ever been used in one other known study where participants reported tobacco ad exposure [9]. In the current study, the images were evaluated for frequency of pro- and anti-tobacco message encounters as well as the characteristics and themes of the messages. This study provides further information not only on how tobacco ads are encountered but also how anti-tobacco messages are encountered, giving tobacco regulators and public health officials a more complete picture of the tobacco messaging environment for low SEP groups.

## Methods

### Overview

Using a systematic, quantitative content analysis, two trained coders examined tobacco-related images uploaded by participants who took part in a mobile health study that aimed to measure the exposure to tobacco messages in a real-world setting and test the feasibility of using this method to gather data on this population. A codebook was developed a priori to examine message type and message characteristics to understand the nature and theme of tobacco-related messages encountered in real-time.

Individuals in low SEP groups (*N* = 95), aged 18–34 years old, who were smokers and non-smokers, from the Boston and Houston areas, took part in a mobile health study. Participants were recruited through community centers using flyers as well as through online advertisements. Participants were asked to report through the app when they encountered a tobacco message in their daily routines. After reporting that they saw a message, they were asked to upload a photo if they were able. They submitted images of tobacco-related messages they encountered (e.g., tobacco advertisements, anti-tobacco messaging) online or in the real world via an application on their mobile phone in real-time for a 7-week period from 2016 to 2017. The application was called Ethica, which participants could download and register on using their email and password on their Android phones. More information on the application can be found elsewhere [21]. Participants were compensated $220 for completing the study.

### Unit of analysis

The unit of analysis was “tobacco message,” which meant that every message was coded separately. In few instances, there was an uploaded image with multiple tobacco messages in that image. Each tobacco message within the image was coded separately.

### Coder training

A codebook was developed a priori to examine message type and message characteristics to understand the nature and theme of tobacco-related messages encountered in real-time. Two coders underwent 5 h of training, which included review of the codebook, coding sample messages, group sample coding messages, and discussions over disagreements on coding. Then, 13 images (10%) of the total sample were coded by each coder to calculate reliability. Reliability ranged from Krippendorff’s alpha = .73 to 1.0, which is within the acceptable range for reliability [27].

### Content categories

The team coded for the following categories: message type (pro- or anti-tobacco), product type, location of message, message source, characteristics of the message, and theme of the message.

#### Message type

Message type was coded as either pro-tobacco or anti-tobacco based on the words and pictures in the images as well as written (e.g., smoking kills, smoke Newports) or implied (e.g., cigarette in the center of a circle with a strike across it, person in image using e-cigarette with smile) [28].

#### Product type

Product type was coded as traditional cigarettes, cigars, cigarillos, or little cigars, smokeless tobacco, e-cigarettes or vape pens, hookah or waterpipe, marijuana (medicinal or recreational), or other. This category was not mutually exclusive [28].

#### Location of message

Location of message was coded as magazine, billboard, outside of a convenience store or gas station, inside or on the inside window of a convenience store or gas station, inside a grocery store or drug store, inside a tobacco store, smoke shop, or hookah bar, bus stop, in a bar or restaurant, in a non-distinct place of business, in a television show or movie, on the Internet, or other [28].

#### Message source

Message source was coded as the following: Industry-sponsored: image was made by an industry organization for tobacco product promotion; User-generated Internet: image was made and uploaded by another user and intended for promotion of tobacco product; Industry-sponsored shared by user: image was made by tobacco industry organization but uploaded or shared by user; Anti-tobacco sponsored: image was made by a public health campaign, health department, government, non-profit etc.; Third party (news, entertainment, etc.) – e.g., New York Times; Other: e.g., cigarette butts on ground, pictures taken by participant of others using tobacco or wearing branded clothing that are not posed or intentional; and Unclear [28].

#### Characteristics of the message

Messages were coded for presence or absence of human beings and cartoons, respectively. Messages were coded as to whether or not the actual product was shown in the message and the number of colors in the message. Pro-tobacco messages were also coded for having a warning label or not [29].

#### Message theme

Pro-tobacco messages were coded for the following themes taken from previous research [29]: 1) conventional themes were defined as focusing on a high-quality product, available at a good economic deal, and for consumer satisfaction; 2) comparative reasons were defined as having a portrayal of product as different (and therefore, less harmful) than other tobacco products; 3) lifestyle factors were defined as portraying their product as an enhancement of users’ lifestyle including bold/lively and glamour/luxury; 4) sex role model endorsement was defined as utilization of masculine or feminine sex role model endorsement theme for the product, including portrayal as masculine or feminine in image or product character; 5) benefits of use was defined as alluding to tobacco use leading to good health and relaxation; 6) social reasons for use was defined in such a way that tobacco use is legitimate because of use by authority figures (such as doctors, lawyers, scientists), and/or due to popularity and use by the common person. Anti-tobacco message themes were coded as: 1) social defined as a message that talks about the social benefits such as not having to leave your friends to smoke or not smelling of tobacco; 2) addiction defined as a message that talks about the risks of addiction to tobacco; 3) health harms defined as a message that talks about the health harms related to using tobacco.

### Data analysis

Descriptive statistical methods including percentages and frequencies were used to analyze the data using SPSS version 28.

## Results

### Message type

Participants reported seeing a total of 484 tobacco messages. Of those, 133 were uploaded as images by 49 participants. There were no significant differences in demographic variables (e.g., age, race/ethnicity, gender, smoking status) between those who uploaded images and those who did not. Participant characteristics can be found in Table 1. One image contained three messages, thus, 136 tobacco messages were coded. Of those messages, 83 (61%) were pro-tobacco, 32 (24%) were anti-tobacco, and 21 (15%) were signage prohibiting smoking in a particular area.

**Table 1:** Sample characteristics, *N* = 49 who uploaded images

	N (%) or mean (SD)
*Age (years)*	24.6 (5.6)
*Race and ethnicity*	
White	14 (28.6)
Black	18 (36.7)
Asian	1 (2.0)
“I am not sure”	14 (28.6)
*Hispanic*	22 (44.9)
*Gender*	
Female	30 (61.2)
Male	16 (32.7)
Prefer not to answer	3 (6.1)
*Smoking status*	
Non-smoker	17 (34.7)
Former smoker	15 (30.6)
Current smoker	14 (28.6)

*N* = 49 participants who uploaded images. For measures without a 49 count, those items were missing

### Pro-tobacco message characteristics

Of the pro-tobacco messages, the majority were for cigarettes (*n* = 67; 80.7%), followed by e-cigarettes (*n* = 10; 12.0%), smokeless tobacco (*n* = 4; 4.8%), and cigarillos, cigars, or little cigars (*n* = 2; 2.4%). One image featured an unclear product. Categories were not mutually exclusive. For example, a sign for a smoke shop could mention more than one product.

The majority of pro-tobacco messages were located outside (36.1%) or inside (30.1%) a convenience store or gas station, followed by a non-distinct place of business (13.3%), a television show, movie, or commercial (4.8%), magazine (3.6%), billboard (1.2%), inside a smoke shop (1.2%), and on the Internet (1.2%). A few (8.4%) were in unclear locations. Most (94.0%) of the messages were industry-sponsored and 4.6% were from a third-party, such as news and entertainment. One was unclear.

Only 7.2% of the pro-tobacco messages featured human beings and only 4.9% had cartoon characters present. The actual product being advertised was only shown in less than a quarter of the messages (24.4%). The majority of pro-tobacco messages (63.9%) contained three to five colors or six or more colors (30.1%). Just over half (51.2%) showed the price of the product. Conventional themes (e.g., high quality, good economic deal) were the most prevalent (53.2%), followed by lifestyle factors (e.g., glamor, living boldly; 22.8%), comparative reasons for use (e.g., freedom from smoking restrictions; 16.5%), sex role models (5.1%), benefits of use (e.g., relaxation; 5.1%), and social reasons (3.8%). Three-fourths (78.2%) had some sort of anti-tobacco warning. It is important to note that all tobacco products under FDA authority were required to carry a warning after these data were collected.

### Anti-tobacco message characteristics

Of the anti-tobacco messages, 75% were for cigarettes, followed by e-cigarettes (12.5%), tobacco in general (9.3%), cigars, cigarillos, or little cigars (6.3%), and smokeless tobacco (3.1%). Categories were not mutually exclusive.

Most messages were located in a television show, movie, or commercial (34.4%), or “other” space such as outdoor parks (18.7%). The next most frequent locations were on the Internet (12.5%), inside a convenience store or gas station (12.5%), in a non-distinct place of business (12.5%), outside a convenience store or gas station (6.3%), or in a bar or restaurant (3.1%). Anti-tobacco message sources were from public health departments, governmental agencies, or non-profits (93.8%), sources such as third parties (i.e., news outlets, etc.; 3.1%), or non-distinct sources (i.e., taking a picture of others with branded clothing; 3.1%).

Almost half of anti-tobacco messages (46.9%) featured human beings that were a plurality male only (40%), followed by both male and female (33.3%), and then female only (26.7%). 43.8% of the anti-tobacco messages contained three to five colors. The most frequently used theme in the anti-tobacco messages was health harms (46.9%), followed by the drawbacks of using tobacco, such as the loss of money or housing due to smoking (6.3%). The rest of the messages had no particular theme.

## Discussion

The current study utilized a content analysis of real-time uploaded tobacco messages by low SEP groups. Results showed that an overwhelming majority of the messages were pro-tobacco and were mostly encountered inside and outside convenience stores and gas stations. These results coincide with previous studies [18–20] and provide more information about real-time exposure to tobacco messages. This study also offers information about how little low SEP groups encounter anti-tobacco messaging.

In the current study’s sample of low SEP groups, results showed that conventional themes (e.g., price) were encountered most frequently. Policy regulations that take into consideration vulnerable groups’ high exposure of these marketing tactics may consider requiring anti-tobacco messaging at these locations to counteract these tactics. Regulations, such prohibiting advertising price reductions or banning point-of-sale advertising, could greatly impact this alluring advertising tactic’s ability to persuade low SEP groups to use tobacco products.

Most interesting of the findings in this study are the infrequent exposures to anti-tobacco messages, and the almost equal exposure to signage prohibiting smoking in a certain space. While exposure to signage prohibiting smoking contributes to the discouragement of use in the social context, it is not enough. Ideally, regulators and public health professionals would want to reach audiences with comprehensive messaging about the risks of smoking and about stopping or avoiding the use of tobacco products and give the tools to do so within the messaging (e.g., quitline information) [24]. In addition, these messages should be part of well-funded and sustained public health campaigns that target all sectors of the community, particularly low SEP groups. Instead, this study showed heavy exposure to signs simply prohibiting smoking.

## Limitations

While this study provided important information, it has its limitations. First, the study was conducted with a small sample of individuals susceptible to selection bias in only two major cities in the U.S. Future studies should expand the sample size and the location of individuals. In addition, some individuals may not have been able to upload every message they saw; such missed images could not be analyzed for theme or content. Therefore, this may not be a complete picture of what tobacco messages individuals in the sample were encountering in their day-to-day life. However, it still does give a more complete picture than requiring participants to recall messages they saw in the past because these data were collected in real-time. Finally, this was from a short period of time. Studies examining exposure over the course of a longer period may provide more extensive information about the tobacco messages encountered. Nonetheless, this study does show that 1) real-time reporting of tobacco messaging exposure can be realized, 2) low SEP groups are encountering a large number of pro-tobacco ads and fewer anti-tobacco messages, and 3) ads were encountered at point-of-sale locations with price reduction tactics. In all, this provides important evidence for regulators of tobacco advertising and public health professionals interested in providing ample and location appropriate anti-tobacco messaging.

## Conclusion

Low SEP groups in this study encountered more pro-tobacco than anti-tobacco messages at places that were point-of-sale. Advertisers frequently use conventional themes, including price promotions to appeal to this group. Future research may consider examining whether anti-tobacco messages at point-of-sale may help combat the advertisements encountered there, especially for price-sensitive, low SEP groups.

This study offers important preliminary evidence for tobacco regulators. It illustrates that low SEP groups are encountering more pro-tobacco messages than anti-tobacco messages. These pro-tobacco messages use tactics focused on price, among a group that may be price sensitive. In addition, this study provides preliminary evidence that low SEP groups, at least in this sample, are not encountering anti-tobacco campaigns at a high rate and may consider more communication programs to help resolve tobacco use disparities. Based on this study, regulators also may consider placing anti-tobacco messaging in locations where pro-tobacco messaging is so prevalent, such as convenience stores and gas stations.

## Data Availability

The datasets used and/or analysed during the current study are available from the corresponding author on reasonable request.

## Abbreviations

SEP: Socio-economic position
FDA: Food and Drug Administration
